# Marginal resection for osteosarcoma with effective neoadjuvant chemotherapy: long-term outcomes

**DOI:** 10.1186/1477-7819-12-341

**Published:** 2014-11-11

**Authors:** Ming Xu, SongFeng Xu, XiuChun Yu

**Affiliations:** Orthopedic Department, General Hospital of Jinan Military Commanding Region, No.25# ShiFan Street, JiNan City, Shandong Prov 250031 China

**Keywords:** Osteosarcoma, Chemotherapy, Adjuvant, Limb salvage, Marginal resection

## Abstract

**Background:**

We report the long-term outcomes of patients with osteosarcoma who underwent effective preoperative chemotherapy and subsequently underwent marginal resection.

**Methods:**

We reviewed the records of 50 patients with osteosarcoma who underwent marginal resection following effective preoperative chemotherapy; 18 were treated with the MMIA (high-dose methotrexate (HD-MTX), adriamycin (ADR), ifosfamide (IFO)) and cisplatin (DDP), and 32 patients were treated with the DIA (DDP, ADR and IFO). protocol. The functions of the affected limb were evaluated using the revised MSTS93 system. The Kaplan-Meier method was used for survival analysis.

**Results:**

After a median follow-up of 5.5 years, the rates were: overall 5-year cumulative survival 61.7%, event-free survival 57.7%, recurrence 8.5%, pulmonary metastases 42.6%, and excellent to good function of the affected limb 57.7%.

**Conclusions:**

Our results showed that marginal resection can be performed in patients with osteosarcoma who obtain clinically favorable responses to chemotherapy. Patients had a good clinical course and there was no negative effect on rates of survival or local recurrence.

## Background

Neoadjuvant chemotherapy and limb preservation is standard treatment for osteosarcoma of the extremities. The classic surgical goal is to create wide surgical margins, but greater excision of structures leads to poorer function. Recently, the need for wide surgical margins has been questioned, with the aim of preserving limb function. Surgical resection should be minimized whenever possible without increasing the risk of local recurrence or jeopardizing the patient’s life. This is the ideal therapeutic strategy for preserving the quality of life of patients with osteosarcoma [1, 2]. Since December 1999, we have treated osteosarcoma with marginal resection when a favorable response to chemotherapy is clinically obtained. The purpose of this study was to evaluate the long-term clinical outcomes of marginal resection for osteosarcoma of the extremities.

## Methods

### Patient and chemotherapy protocol

Between December 1999 and October 2008, 50 patients with osteosarcoma (28 male, 22 female; average age of 17 years) underwent marginal resection following effective preoperative chemotherapy. The distal femur was involved in 24 patients, proximal tibia in 19, proximal humerus in 4, proximal fibula in 2, and proximal femur in 1. Of the 50 patients, 49 patients had stage IIB, and 1 had stage IIIB cancer. Chemotherapy drugs comprised high-dose methotrexate (HD-MTX 10 g/m^2^), adriamycin (ADR 90 mg/m^2^), ifosfamide (IFO 2.0 g/m^2^ × 5), and cisplatin (DDP 120 mg/m^2^), given as one of two chemotherapy protocols: the MMIA protocol (HD-MTX, ADR and IFO) and the DIA protocol (DDP, ADR and IFO). Of the 50 patients, 32 received DIA and 18 received MMIA. Before surgery, Since the 1980s, osteosarcoma has commonly been treated with neoadjuvant chemotherapy. 6 to 10 courses of chemotherapy were performed.

### Evaluation of the chemotherapeutic response

The effectiveness of chemotherapy was evaluated before surgery by clinical evidence, laboratory tests, plain radiography and magnetic resonance imaging (MRI). Evidence of a good chemotherapeutic response included alleviation of pain, shrinkage and hardening of the local mass, improved range of motion in the affected limb; reduction in serum alkaline phosphatase (ALP) and lactate dehydrogenase (LDH) concentrations; sclerotic changes and clearly defined lesion margins observed on plain radiographs; and marked shrinkage of the tumor, reduction in marrow edema, and shrinkage of tumor extension into soft tissue observed on MRI.

**Figure 1:**
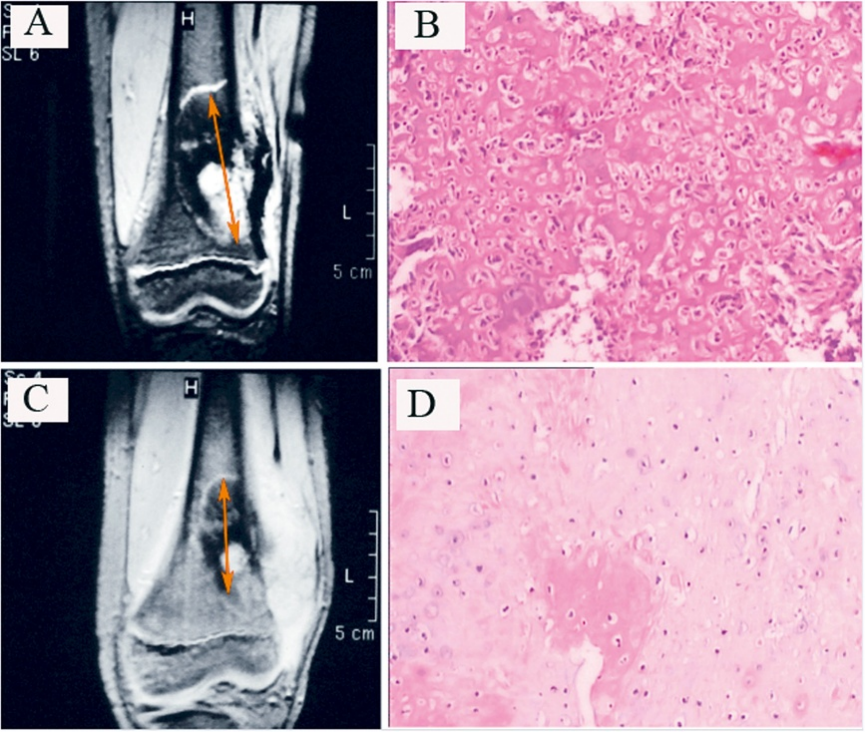
**Osteosarcoma arising from the left distal femur in a 15-year-old boy. (A,B)** Before chemotherapy. **(A)** Magnetic resonance imaging (MRI) scan of the tumor obtained before neoadjuvant chemotherapy; arrows show lesions in longest diameter. **(B)** Tumor cells (hematoxylin and eosin (H&E), original magnification × 40). **(C,D)** After chemotherapy. **(C)** MRI scan of the same tumor after chemotherapy showed that there was no tumor extension into the epiphysis. Extensive calcification and the significantly reduced lesion (arrows, the sum of the longest diameter ≥30% reduction) indicated a good response to therapy. **(D)** Significant tumor cell degeneration and necrosis (H&E, original magnification × 40).

The grade of tumor cell necrosis rate (TCNR) was classified according to the Huvos necrosis grading system [3]: grade I, necrosis rarely seen; grade II, <50% and ≤90% tumor necrosis; grade III, >90% and <100% necrosis; and grade IV, 100% necrosis. Grades I and II were defined as poor responses, and grades III and IV as good responses (Figure 1).

### Surgical procedure

**Figure 2:**
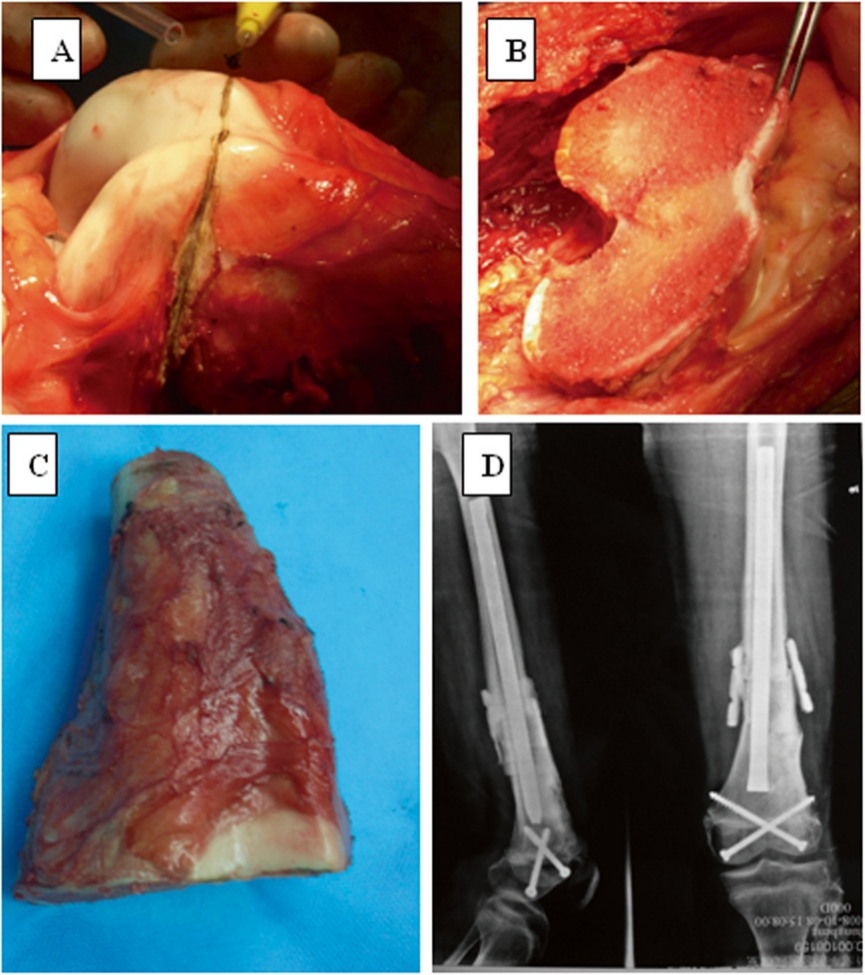
**Results for a patient treated for osteosarcoma. (A)** The epiphysis of the distal femur was preserved by marginal resection; **(B)** the remaining epiphysis; and **(C)** the resected tumor. **(D)** The radiograph shows that after an intramedullary nail and screws had been inserted into the residual epiphysis and the bone defect filled with bone cement (no barium), the implanted bone healed successfully.

Limb-salvaging surgery was conducted by means of intentional marginal resection, defined as the plane of dissection through the pseudocapsule or peritumoral reactive tissue. This technique preserves important structures such as the major neurovascular bundles, tendons, ligaments, muscles, and the epiphysis. Of the 50 patients, 28 underwent tumor resection and implantation of autoclaved bone graft. For these patients, the possibility for preservation of the epiphysis was compatible with the MRI findings. The epiphysis was preserved in six (Figure 2).

Of the remaining 22 patients, 17 underwent tumor resection and prosthetic replacement, 3 underwent tumor resection with autograft implantation, and 2 patients with involvement of the proximal fibula underwent marginal tumor resection of the fibula head, preserving the common peroneal nerve, lateral collateral ligament, and biceps femoris tendon.

### Observation and follow-up

After completion of chemotherapy, patients were examined by radiography of the operated limb and computed tomography of the chest every 3 months for 2 years, then at at 6-month intervals for another 3 years. Recurrence, metastasis, and death were recorded. The functions of the affected limb were evaluated using the revised 30-point functional classification system established by the International Society of Limb Salvage and the Musculoskeletal Tumor Society (MSTS93) [4].

### Statistical analysis

The Kaplan-Meier method was used for survival analysis. All statistics were performed with SPSS software (version 20.0; SPSS, Chicago, IL, USA) and *P* <0.05 was regarded as significant.

## Results

### Patient characteristics

All 50 patients completed preoperative chemotherapy. The histological response to preoperative chemotherapy was grade IV in 27 patients, grade III in 16, and grade II in 7. All patients also underwent postoperative chemotherapy. Of the 50 patients, 19 were unable to complete planned chemotherapy; 16 of these were due to early progression, and 3 because of lack of funds. Of the remaining 31 patients, 26 received all 12 cycles of chemotherapy, 3 received 11 cycles, and 2 received 10 cycles. A total of 455 cycles of chemotherapy were carried out for the 50 patients.

### Complications

During follow-up, four patients underwent reoperation. One patient underwent open reduction, bone grafting, and internal fixation because of an inactivated bone fracture at 26 months after the initial surgery; the bone had healed well by 4 months after reoperation. At 100 months after the second operation, the patient was able to walk without orthopedic footwear, but the affected limb was 2 cm shorter than the unaffected limb. Flexion of the affected knee joint was 110° with a limb function score of 28.

The second patient required reoperation 18 months after the first operation because of inactivated bone fracture. At 42 months after the first operation, this patient was able to walk normally. The affected limb was 1 cm shorter than the unaffected limb and flexion of the affected knee joint was 135°, with a limb function score of 30.

The thrid patient underwent open reduction and allografting combined with autografting for inactivated bone fracture 12 months after the initial surgery.

The fourth patient underwent arthroscopic release, quadricepsplasty, and continuous passive motion for knee stiffness at 3 years after the initial operation. At the last follow-up, 89 months after the initial operation, the length of both legs was equal, and the affected knee had normal flexion.

### Survival analysis

Living patients were followed clinically for a minimum of 5 years. The median follow-up was 5.5 years (range 8 to 160 months) for all patients, and 7.3 years (range, 60 to 160 months) for living patients. Three patients were lost to follow-up.

Four patients had local recurrence (time lapse ranged from 6 to 21 months after surgery) and two of them underwent amputation; the local recurrence rate for this group was 8.5% (4/47). Pulmonary metastases developed in 20 patients (42.6%) during follow-up; 19 died and 1 is currently alive with tumor.

**Figure 3:**
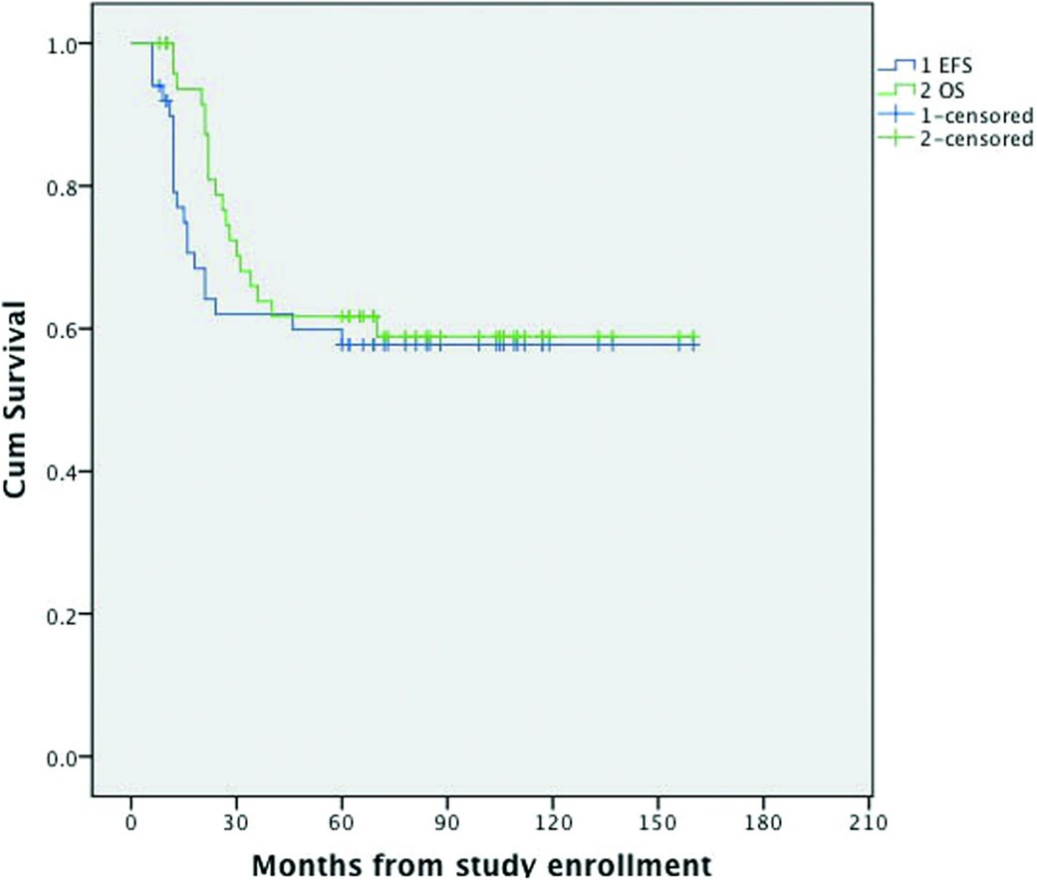
**Overall survival and event-free survival of patients with osteosarcoma treated in our hospital.**

For the full patient group, the overall 5-year survival rate was 61.7%, and the event-free survival rate was 57.7% (Figure 3).

### Functions

Functional evaluation of these patients was classified according to MSTS criteria: limb function was excellent in 24 patients, good in 12, fair in 10 and poor in 4. The combined excellent and good rate for affected limbs was 72%. Of the six patients who underwent limb-salvage operations that preserved the epiphysis, three could flex the affected knee joint to 110°, two could flex 90° to 110°, and one could flex 60° to 90°. The length of the affected limb equaled that of the unaffected limb in four patients, and was 2 cm shorter than the other limb in two patients.

## Discussion

The current standard treatment for osteosarcoma includes neoadjuvant chemotherapy and limb-sparing surgery. Currently, two methods of tumor resection exist: wide resection and marginal resection. This surgical staging system proposed by Enneking *et al*. [5] incorporates margin definitions predicting for local recurrence based on the relationship of the surgical margin to the neoplasm and its surrounding pseudocapsule and reactive zone. Wide resection is defined as removal of the gross tumor with at least 3 cm of normal surrounding soft tissue and 5 cm of normal surrounding bone tissue. Wide resection often requires excising more normal soft and bone tissue around the osteosarcoma, leading to a higher rate of operative complications and poorer limb function. Marginal resection, defined as plane of dissection through the pseudocapsule or peritumoral reactive tissue, removes the gross tumor with 1 cm of normal surrounding soft tissue and 2 to 3 cm of normal surrounding bone tissue. Marginal resection can preserve the surrounding areas that are normally resected, such as ligaments, nerves, blood vessels, and the epiphysis region. Marginal resection of osteosarcoma should be performed only if the preoperative chemotherapeutic effect has been rated as a good response.

The aims of marginal resection of osteosarcoma are to reduce bone and soft-tissue defects and to improve limb function, without increasing the risk of local recurrence or jeopardizing the patient’s life. Since 1987 to 2004, Tsuchiya’s group have treated 21 patients with osteosarcoma with intentional marginal resection following good results from chemotherapy [1, 2, 6]. Four patients with lesions in the proximal fibula were treated by marginal resection of the fibular head, which preserved the common peroneal nerve, lateral collateral ligament, and biceps femoris tendon. The epiphysis was preserved in eight patients, and the bone defect was reconstructed by distraction osteogenesis. Joint resection (resection of one or both epiphyses) was performed in nine patients. No local tumor recurrence was seen during a mean follow-up of 97 months. Pulmonary metastases developed in four patients during a a mean follow-up of 35.8 months. The overall cumulative survival rate was 100% and the event-free survival rate was 78%. The overall average function of the 21 patients was evaluated as 93% of normal. Thus, this group considered that it was possible to treat osteosarcoma by marginal resection along with good preoperative chemotherapy.

Between December 1999 and October 2008, we treated osteosarcoma with marginal resection [7], when a favorable response to chemotherapy was clinically obtained. During this period, 50 patients underwent marginal resection and were followed up for a mean time of 5.5 years. Four patients had a local recurrence with time lapse ranging from 6 to 21 months after surgery (local recurrence rate 8.5%). Pulmonary metastases developed in 20 patients (42.6%) during follow-up. The overall 5-year survival rate was 61.7%, and the event-free survival rate was 57.7%. We consider that marginal resection is an option for osteosarcoma with good response to preoperative chemotherapy, because our data are similar to those reported by others [8–12].

Effect of chemotherapy and size of surgical margin are the most important factors for osteosarcoma prognosis, and Tsuchiya *et al*. suggested that the former is more important than the latter for limb-salvaging procedures [1]. Efficient chemotherapy improves prognosis and minimizes surgical margins. Thus, when a smaller resection margin is being considered, it is most important to confirm the effectiveness of chemotherapy and the accuracy of preoperative assessment of the extent of intramedullary tumor. When a good response is documented by at least two of four radiological methods, including plain radiography, MRI, angiography, and 201Ti scintigraphy, more than 90% tumor necrosis can be expected and minimized resection can be performed [6]. We confirmed preoperative chemotherapeutic effects not only by plain radiography and MRI but also by changes in clinical appearance and serum LDH and ALP concentrations [13]. We considered that the following provided evidence of good chemotherapeutic response: reduction of pain; shrinkage and rigidity of any local mass; normal range of movement of the affected limb; reduction of serum ALP and LDH to within the normal range; sclerotic changes and clearly defined lesion margins visible on plain radiographs; and marked shrinkage of tumor, reduction of marrow edema, and marked shrinkage of any extension of the tumor into soft tissue on MRI. If these did not occur, the chemotherapeutic response was graded as poor.

## Conclusions

Our results showed that marginal resection should be performed by an experienced surgeon who is familiar with handling limb salvage in cases of malignant bone tumor. In conclusion, no adverse effects on survival or local recurrence were found, and our outcomes indicate that it is possible to perform marginal limb-sparing surgery in conjunction with effective chemotherapy for limb osteosarcoma.

## Authors’ information

MX, SX, and XY are staff members of Orthopedic Department, General Hospital of Jinan Military Commanding Region.

## Abbreviations

HD-MTX: high-dose methotrexate
ADR: adriamycin
IFO: ifosfamide
DDP: cisplatin
MMIA: high-dose methotrexate, adriamycin, ifosfamide
DIA: cisplatin, ifosfamide, adriamycin
MRI: magnetic resonance imaging
ALP: alkaline phosphatase
LDH: lactate dehydrogenase
TCNR: tumor cell necrosis rate.

## References

[1] Tsuchiya H, Tomita K, Mori Y, Asada N, Yamamoto N (1999). Marginal excision for osteosarcoma with caffeine assisted chemotherapy. Clin Orthop Relat Res.

[2] Kanazawa Y, Tsuchiya H, Nonomura A, Takazawa K, Yamamoto N, Tomita K (2003). Intentional marginal excision of osteosarcoma of the proximal fibula to preserve limb function. J Orthop Sci.

[3] Huvos AG (1997). Bone tumors: Diagnosis, treatment, and prognosis.

[4] Enneking WF, Dunham W, Gebhardt MC, Malawar M, Pritchard DJ (1993). A system for the functional evaluation of reconstructive procedures after surgical treatment of tumors of the musculoskeletal system. Clin Orthop Relat Res.

[5] Enneking WF, Spanier SS, Goodman MA (1980). A system for the surgical staging of musculoskeletal sarcoma. Clin Orthop Relat Res.

[6] Hayashi K, Tsuchiya H, Yamamoto N, Takeuchi A, Tomita K (2008). Functional outcome in patients with osteosarcoma around the knee joint treated by minimised surgery. Int Orthop.

[7] Yu XC, Xu M, Song RX, Xu SF (2009). Marginal resection for osteosarcoma with effective preoperative chemotherapy. Orthop Surg.

[8] Picci P, Mercuri M, Ferrari S, Alberghini M, Briccoli A, Ferrari C, Pignotti E, Bacci G (2010). Survival in high-grade osteosarcoma: improvement over 21 years at a single institution. Ann Oncol.

[9] Daw NC, Neel MD, Rao BN, Billups CA, Wu J, Jenkins JJ, Quintana J, Luchtman-Jones L, Villarroel M, Santana VM (2011). Frontline treatment of localized osteosarcoma without methotrexate. Cancer.

[10] Assi H, Missenard G, Terrier P, Le Pechoux C, Bonvalot S, Vanel D, Meric JB, Tursz T, Lecesne A (2010). Intensive induction chemotherapy without methotrexate in adult patients with localized osteosarcoma: results of the Institut Gustave-Roussy phase II trial. Curr Oncol.

[11] Ferrari S, Smeland S, Mercuri M, Bertoni F, Longhi A, Ruggieri P, Alvegard TA, Picci P, Capanna R, Bernini G, Müller C, Tienghi A, Wiebe T, Comandone A, Böhling T, Del Prever AB, Brosjö O, Bacci G, Saeter G, Italian and Scandinavian Sarcoma Groups (2005). Neoadjuvant chemotherapy with high-dose Ifosfamide, high-dose methotrexate, cisplatin, and doxorubicin for patients with localized osteosarcoma of the extremity: a joint study by the Italian and Scandinavian Sarcoma Groups. J Clin Oncol.

[12] Fuchs N, Bielack SS, Epler D, Bieling P, Delling G, Körholz D, Graf N, Heise U, Jürgens H, Kotz R, Salzer-Kuntschik M, Weinel P, Werner M, Winkler K (1998). Long-term results of the co-operative German-Austrian-Swiss osteosarcoma study group’s protocol COSS-86 of intensive multidrug chemotherapy and surgery for osteosarcoma of the limbs. Ann Oncol.

[13] Bacci G, Longhi A, Ferrari S, Briccoli A, Donati D, De Paolis M, Versari M (2004). Prognostic significance of serum lactate dehydrogenase in osteosarcoma of the extremity: experience at Rizzoli on 1421 patients treated over the last 30 years. Tumori.

